# The Microbiota–Gut–Brain Axis Across the Lifespan: From Neurodevelopment to Neurodegeneration

**DOI:** 10.3390/jcm15083065

**Published:** 2026-04-17

**Authors:** Salvatore Michele Carnazzo, Vassilios Fanos

**Affiliations:** 1 Department of Medicine and Surgery, University of Enna “Kore”, 94100 Enna, Italy; 2 Neonatal Intensive Care Unit, AOU Cagliari, Department of Surgical Sciences, University of Cagliari, 09124 Cagliari, Italy; vafanos@jpnim.com

**Keywords:** microbiota–gut–brain axis, neurodevelopment, neurodegeneration, inflammation, short-chain fatty acids, Alzheimer’s disease, Parkinson’s disease

## Abstract

The microbiota–gut–brain axis (MGBA) is a complex bidirectional communication network integrating neural, endocrine, immune, and metabolic pathways linking intestinal microbiota to central nervous system function. Increasing evidence indicates that microbiota-derived signals are critical regulators of neurodevelopment and may contribute to vulnerability to neurodegenerative disorders across the lifespan. In this narrative review, we synthesize experimental and clinical evidence to define the key biological mechanisms underlying microbiota–brain interactions. Converging data indicate that immune activation, barrier dysfunction, and microbial metabolites, particularly short-chain fatty acids and tryptophan-derived compounds, represent central mediators linking gut dysbiosis to neuroinflammatory and neurodegenerative processes. Early-life microbial perturbations, driven by factors such as antibiotic exposure, diet, and psychosocial stress, appear to induce long-term immunometabolic programming that may increase susceptibility to neurological disorders later in life. Clinical studies consistently associate dysbiosis with neurodevelopmental conditions and major neurodegenerative diseases, including Alzheimer’s disease and Parkinson’s disease; however, causal relationships remain incompletely defined due to heterogeneity and the predominance of observational data. Overall, the available evidence supports a lifespan model in which microbiota-driven immune and metabolic dysregulation contributes to both early neurodevelopmental trajectories and late-life neurodegeneration. While microbiome-based biomarkers and therapeutic strategies show promise, their clinical translation requires validation in longitudinal and interventional studies.

## 1. Introduction

The microbiota–gut–brain axis (MGBA) refers to a complex bidirectional communication network linking the intestinal microbiota with the gastrointestinal tract, the enteric and autonomic nervous systems, and central nervous system circuits that regulate physiology, cognition, and behavior [1]. Within this framework, the gut microbiota functions as an active endocrine, metabolic, and immune signaling hub capable of generating molecular signals that influence brain function through neural, immune, endocrine, and metabolic pathways [2]. Experimental and clinical evidence increasingly indicates that microbial signals influence fundamental neurobiological processes, including microglial maturation, blood–brain barrier integrity, myelination, and neurogenesis, thereby shaping behavioral and cognitive outcomes across the lifespan [2,3]. These interactions are highly dependent on developmental timing. Early life represents a critical window during which microbial colonization coincides with immune maturation and brain development, allowing microbial signals to influence long-term neurobiological trajectories [2,3]. At the opposite end of the lifespan, age-related alterations in microbiome composition and metabolic activity may contribute to systemic inflammation, barrier dysfunction, and neuroimmune dysregulation, processes increasingly implicated in neurodegenerative disorders [2]. These observations suggest that microbiota-dependent signaling pathways represent potential mechanistic links between early neurodevelopmental programming and vulnerability to late-life neurodegenerative disorders. In this review, we examine the microbiota–gut–brain axis from a lifespan perspective, integrating evidence from experimental models, human cohort studies, and mechanistic research, including an overview of the principal biological pathways mediating microbiota–brain communication, a discussion of the role of early-life microbial colonization in neurodevelopmental programming and of the mechanistic bridges that may connect early microbial perturbations with neurodegenerative vulnerability later in life, and finally an examination of environmental and lifestyle factors that modulate microbiota–brain interactions and their emerging translational implications for prevention and therapeutic intervention. From this perspective, neurodegenerative disorders may not represent isolated late-life events, but rather the long-term consequence of microbiota-driven immune and metabolic programming occurring across the lifespan.

## 2. Literature Search Strategy

This narrative review is based on a non-systematic, iterative literature search conducted using PubMed, Scopus, and Web of Science databases. Multiple targeted search strategies were applied to capture key domains of the microbiota–gut–brain axis across the lifespan, including neurodevelopment, neuroinflammation, environmental exposures, and neurodegeneration. Search queries combined terms related to gut microbiota (e.g., “microbiome”, “dysbiosis”), brain development and function (e.g., “neurodevelopment”, “microglia”, “blood–brain barrier”), early-life exposures, and neurodegenerative processes (e.g., “Alzheimer’s disease”, “Parkinson’s disease”). Studies were selected based on relevance, methodological quality, and contribution to mechanistic understanding, including both experimental and clinical evidence. Reference lists of key articles were also screened to identify additional relevant studies. No formal systematic review protocol was applied; however, studies were prioritized based on relevance to the microbiota–gut–brain axis, methodological quality, and contribution to mechanistic understanding, ensuring a balanced representation of experimental and clinical evidence.

## 3. Core Pathways of Microbiota–Gut–Brain Communication

**Figure 1:**
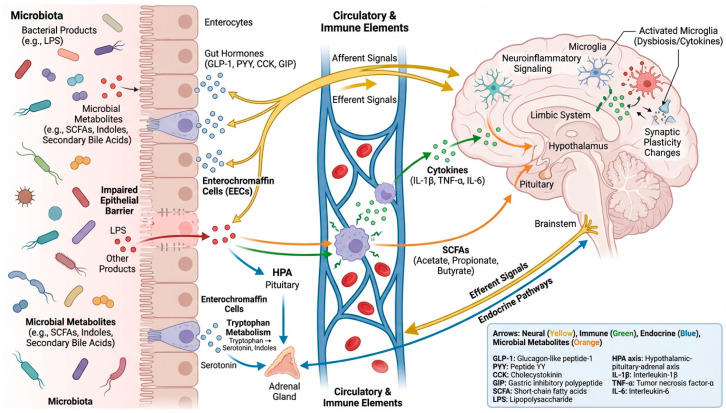
Schematic representation of the microbiota–gut–brain axis (MGBA). The figure illustrates the main bidirectional communication pathways between the gut microbiota and the central nervous system, including neural signaling (vagal pathways), immune mechanisms (cytokine-mediated responses), endocrine regulation (hypothalamic–pituitary–adrenal axis), and microbial metabolites (e.g., short-chain fatty acids and tryptophan-derived compounds). Established pathways are distinguished from emerging or hypothetical mechanisms based on the strength of current evidence. The red arrow represents the translocation of bacterial products (e.g., lipopolysaccharide, LPS) and other microbial components across an impaired intestinal epithelial barrier into the systemic circulation.

Bidirectional communication along the microbiota–gut–brain axis is mediated by multiple interacting pathways integrating neural, endocrine, immune, and metabolic signaling mechanisms. The main pathways of microbiota–gut–brain communication are summarized in Figure 1. Neural pathways represent one of the most rapid routes through which gut-derived signals can influence central nervous system activity. In particular, the vagus nerve functions as a major conduit for gut–brain communication, integrating sensory information derived from microbial metabolites, nutrients, and gut hormones and transmitting these signals to brainstem and limbic circuits involved in behavioral and metabolic regulation [1,2,4,5]. Enteroendocrine cells serve as key intermediaries in this process, detecting luminal stimuli and releasing hormones such as GLP-1, PYY, CCK, and GIP that can activate vagal afferents and influence central appetite and metabolic control [5]. Endocrine signaling constitutes a second major communication route, linking microbial activity with host neuroendocrine responses. The hypothalamic–pituitary–adrenal (HPA) axis plays a central role in this interaction, as microbiota depletion has been associated with exaggerated stress responses in germ-free models, highlighting the importance of microbial signals in regulating neuroendocrine stress reactivity [1,2,6]. In parallel, gut-derived serotonin produced by enterochromaffin cells is strongly influenced by microbial metabolism of tryptophan, thereby linking microbial ecology with host serotonergic signaling pathways [5,7]. Immune and barrier-mediated pathways represent another critical mechanism of gut–brain communication. Dysbiosis can impair intestinal epithelial integrity, facilitating the translocation of bacterial products such as lipopolysaccharide (LPS) into systemic circulation and triggering innate immune activation with increased cytokine production, including IL-1β, TNF-α, and IL-6, which can promote neuroinflammatory responses and alter synaptic plasticity [4,6,8]. Within the central nervous system, microglia function as a key integrative node linking peripheral immune signals to neural outcomes, and studies in germ-free animals demonstrate altered microglial maturation that can be restored by microbial metabolites such as short-chain fatty acids (SCFAs) [2,3,9]. Finally, metabolic signaling mediated by microbial metabolites provides an additional interface between gut microbiota and brain physiology. SCFAs, including acetate, propionate, and butyrate, influence host physiology through receptor-mediated signaling and epigenetic regulation, contributing to barrier integrity, immune homeostasis, and neuroinflammatory modulation [2,5,9]. Additional metabolite pathways, such as microbial regulation of tryptophan metabolism and the production of indoles and secondary bile acids, generate neuroactive molecules capable of influencing immune signaling, neurotransmission, and neuronal vulnerability [10]. Together, these interconnected pathways form an integrated communication network through which microbial signals can influence brain development, immune regulation, and neurological function across the lifespan.

## 4. Early-Life Microbiota and Neurodevelopmental Programming

Early life represents a critical developmental window during which gut microbiota assembly, immune maturation, and central nervous system development occur in parallel, rendering the host particularly sensitive to microbial perturbations and their downstream immunometabolic consequences. Microbial colonization during infancy is strongly influenced by delivery mode, early feeding practices, and antibiotic exposure, which shape microbiota succession and immune education with potential long-term effects on neurodevelopmental trajectories [11,12]. Foundational experimental studies demonstrate that commensal microbiota are required for normal brain development: germ-free mice show altered anxiety-related behavior, dysregulated monoamine metabolism, and changes in synaptic and neuroplasticity markers, while early, but not adult, colonization partially normalizes these phenotypes, supporting the existence of critical windows for microbiota-dependent neurodevelopmental programming [13]. Mechanistically, microbial metabolites and immune signals influence key neurodevelopmental processes including microglial maturation, synaptic pruning, and neural circuit formation. Alterations in tryptophan-derived microbial metabolites have been linked to microglial activation, synaptic remodeling, and behavioral changes in experimental models, supporting the existence of a microbiota–metabolite–microglia signaling axis during early development [14]. Maternal microbial signals may also influence fetal brain development, as maternal microbiome depletion disrupts fetal axonogenesis and thalamocortical circuit formation, effects that can be reversed by microbial metabolite supplementation [15]. In addition to synaptic development, early microbial status modulates myelination processes. Germ-free models demonstrate altered myelin-related gene expression in the prefrontal cortex that is only partially restored by delayed microbial colonization, suggesting that microbial signals regulate myelin development within defined developmental windows [16]. Perturbations of early microbial communities can also produce long-term neuroimmune and barrier alterations: neonatal antibiotic-induced dysbiosis results in persistent microbiota changes, intestinal barrier dysfunction, and behavioral alterations that can be rescued by supplementation with microbial metabolites such as butyrate derivatives [17]. Early-life dysbiosis may further exacerbate neurodevelopmental vulnerability through inflammatory gut–brain signaling. Experimental models of neonatal brain injury demonstrate that microbial imbalance promotes intestinal barrier disruption, systemic inflammation, and hippocampal neuroinflammation, while microbiota restoration strategies can improve neurocognitive outcomes [18]. Human studies provide convergent evidence supporting these mechanisms. Prospective birth cohorts have identified associations between infant microbiome composition, cord blood metabolomic profiles, and later neurodevelopmental diagnoses including autism spectrum disorder and ADHD, suggesting that microbial and metabolic alterations may precede clinical symptom onset [19]. Similarly, longitudinal studies in extremely preterm infants reveal links between dysbiosis, pro-inflammatory immune activation, and early brain injury, illustrating the close coupling between gut microbial ecology, immune maturation, and neurodevelopment [20]. Finally, early-life interventions targeting microbial colonization after caesarean delivery show variable results: vaginal microbiota transfer may accelerate microbiota maturation and associate with improved early developmental outcomes, whereas other seeding approaches demonstrate limited microbial engraftment, highlighting the complexity of neonatal microbiota assembly [21,22,23]. Complementary interventions, including breastfeeding and early caregiving practices such as skin-to-skin contact, further influence microbiota development and immune maturation, supporting the concept that early environmental exposures shape microbiota–gut–brain signaling pathways with potential long-term implications for neurodevelopmental health [24,25].

## 5. Mechanistic Bridges Across the Lifespan

Accumulating evidence suggests that early-life microbial signals may exert long-lasting effects on brain health by shaping immune, metabolic, and neurovascular pathways that later converge on neurodegenerative vulnerability. Across the lifespan, dysbiosis-driven alterations in gut–brain communication contribute to chronic inflammation, barrier dysfunction, and maladaptive neuroimmune priming, thereby increasing susceptibility to disorders such as Alzheimer’s disease and Parkinson’s disease [26,27]. A central mechanism linking dysbiosis to neurodegeneration is the establishment of chronic low-grade neuroinflammation. Microbial perturbations influence the maturation and responsiveness of innate immune cells, particularly microglia, which require microbial-derived signals for proper immune calibration. Experimental studies show that germ-free conditions alter microglial morphology and cytokine responsiveness, while dysbiosis can activate inflammasome pathways such as NLRP3, promoting caspase-1 activation and the release of IL-1β and IL-18 that amplify neuroinflammatory cascades [26,27,28]. Gut barrier disruption represents another key bridge mechanism. Dysbiosis can impair epithelial tight junction integrity, increasing intestinal permeability and enabling systemic dissemination of microbial products such as lipopolysaccharide (LPS). Even low-grade endotoxemia induces systemic cytokine responses, including TNF-α and IL-6, thereby priming CNS immune signaling and contributing to neuroinflammation [27]. Clinical observations further support this pathway, as elevated circulating LPS and barrier injury markers correlate with increased blood–brain barrier (BBB) permeability and reduced levels of microbial metabolites such as short-chain fatty acids (SCFAs) in inflammatory neurological conditions [29]. Microbial metabolites represent an additional mechanistic interface linking gut dysbiosis to neurodegenerative processes. SCFAs, including acetate, propionate, and butyrate, support epithelial and BBB tight junction integrity, limit endotoxin translocation, and suppress inflammatory signaling, with experimental studies showing that SCFA supplementation can attenuate neuroinflammation and restore barrier function under systemic inflammatory stress [30,31]. Dysbiosis-associated alterations in tryptophan metabolism also contribute to neuronal vulnerability: inflammatory activation of the indoleamine-2,3-dioxygenase pathway shifts tryptophan metabolism toward kynurenine derivatives, increasing neurotoxic metabolites such as quinolinic acid while reducing neuroprotective kynurenic acid, thereby promoting excitotoxicity and oxidative stress implicated in Alzheimer’s and Parkinson’s disease pathogenesis [32]. Additional microbial metabolites, including secondary bile acids, further modulate inflammatory signaling and BBB stability through receptor-mediated pathways involving FXR and TGR5 [33]. Finally, neural communication through the vagus nerve provides a rapid anatomical route linking microbial activity to central immune regulation: microbial metabolites, enteroendocrine hormones, and inflammatory signals can activate vagal afferents, while efferent vagal signaling engages the cholinergic anti-inflammatory reflex via α7 nicotinic receptors, limiting macrophage cytokine production and contributing to barrier preservation under inflammatory stress [34]. These interconnected immune, barrier, metabolic, and neural mechanisms illustrate how microbiota-driven perturbations can progressively shape neuroinflammatory tone and neuronal vulnerability across the lifespan, providing a mechanistic framework linking early microbial dysregulation to late-life neurodegenerative disease. However, direct longitudinal evidence in humans linking early-life dysbiosis to late-life neurodegeneration remains limited, and these relationships should be interpreted with caution.

## 6. Environmental Modulators and Lifespan Vulnerability

Environmental factors represent major modulators of microbiota–gut–brain axis dynamics and may shape neurodevelopmental trajectories and vulnerability to brain disorders across the lifespan. Early life constitutes a particularly sensitive window during which microbial colonization, immune maturation, and neural circuit development occur in parallel, rendering the system highly responsive to environmental perturbations. In this context, environmental exposures can influence microbiome assembly and host immune programming, thereby modulating microbiota–brain communication pathways with potential long-term consequences for brain health. Among the most influential modulators are antibiotic exposure, dietary patterns, psychosocial stress, and environmental toxicants, all of which can induce persistent alterations in microbiome composition and downstream immune–metabolic signaling pathways. Antibiotic exposure during infancy is one of the most potent disruptors of microbiome assembly, and epidemiological studies report modest but consistent associations between prenatal or early-life antibiotic use and later neurodevelopmental disorders such as autism spectrum disorder and attention-deficit/hyperactivity disorder, although familial confounding likely contributes to these associations [35]. Clinical observations in preterm infants further suggest that antibiotic-driven dysbiosis may influence early brain maturation, as alterations in neonatal microbiome composition have been linked to abnormal electrophysiological activity and MRI biomarkers of brain injury [36,37]. These findings illustrate how early perturbations of microbiome development may influence immune maturation and neurodevelopmental trajectories. Diet represents another dominant ecological force shaping microbial ecosystems throughout life. Western dietary patterns characterized by high fat and refined sugar intake promote reduced microbial diversity, intestinal permeability, and inflammatory signaling, whereas fiber-rich Mediterranean-style diets support microbial diversity and the production of beneficial metabolites such as short-chain fatty acids (SCFAs), which can modulate immune responses and neuroinflammation [38,39]. Importantly, SCFAs exert their effects through multiple molecular mechanisms, including activation of G-protein-coupled receptors (FFAR2/FFAR3), inhibition of histone deacetylases, and modulation of immune, endocrine, and neural pathways, thereby influencing gene expression, microglial activation, and blood–brain barrier integrity [40]. In addition, SCFAs can cross the blood–brain barrier and directly influence central nervous system function, further supporting their role as key mediators of microbiota–brain communication [40]. Maternal diet during pregnancy may therefore influence offspring neurodevelopment through microbiome-mediated immune and metabolic pathways, highlighting diet as a potentially modifiable factor shaping microbiota–brain interactions from early life onward. Psychosocial stress represents another environmental regulator of microbiome–brain signaling through its interaction with the hypothalamic–pituitary–adrenal (HPA) axis. Experimental studies demonstrate that stress can induce persistent dysbiosis, inflammatory activation, and exaggerated glucocorticoid responses, while microbial metabolites and immune mediators influence stress-related neural circuits and behavioral outcomes [41,42]. These effects are mediated by alterations in microbial composition that influence immune signaling, cytokine production, and vagal pathways, thereby modulating central stress circuits and behavioral responses [43]. These bidirectional interactions illustrate how neuroendocrine and microbial pathways jointly regulate brain–gut communication. Finally, emerging evidence suggests that environmental toxicants, including air pollution, may also influence brain development through microbiome-mediated mechanisms. Experimental models show that developmental exposure to air pollutants can induce cognitive and behavioral abnormalities accompanied by microbiome alterations, with antibiotic depletion of gut microbes attenuating these effects, supporting a causal microbiome-dependent pathway [44]. Collectively, these findings indicate that environmental exposures interact dynamically with host immune and metabolic systems to shape microbiota–gut–brain axis signaling across the lifespan. Through their effects on microbiome composition, immune regulation, and metabolic pathways, environmental factors may amplify neuroinflammatory processes and barrier dysfunction, thereby contributing to long-term vulnerability to neurodegenerative disorders. In addition to environmental modulators, emerging pharmacological interventions targeting metabolic pathways may also influence microbiota–gut–brain axis signaling. Glucagon-like peptide-1 (GLP-1) receptor agonists, such as semaglutide, have demonstrated neuroprotective effects through the modulation of neuroinflammatory processes, oxidative stress, and neuronal survival pathways. These effects are mediated by key intracellular signaling cascades, including PI3K/Akt, cAMP/PKA, and MAPK pathways, which regulate synaptic function, energy metabolism, and cellular resilience [45,46]. Emerging evidence suggests that GLP-1 receptor agonists may also modulate gut microbiota composition and metabolic activity, thereby potentially influencing microbiota–gut–brain axis signaling, although the current evidence is largely derived from preclinical studies and remains to be fully elucidated.

## 7. Microbiota and Neurodegeneration

**Figure 2:**
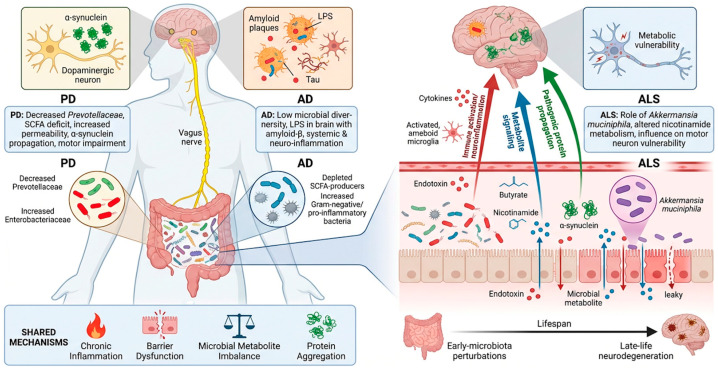
Microbiota–gut–brain axis and shared mechanisms in neurodegenerative diseases. The figure illustrates common microbiota-associated pathways implicated in Parkinson’s disease (PD), Alzheimer’s disease (AD), and amyotrophic lateral sclerosis (ALS), including dysbiosis, intestinal barrier dysfunction, endotoxin translocation, and immune activation. Microbial metabolites such as short-chain fatty acids and nicotinamide, together with vagal and systemic signaling pathways, contribute to neuroinflammation, protein aggregation (e.g., α-synuclein, amyloid-β, tau), and neuronal vulnerability across the lifespan. Established pathways are distinguished from emerging or hypothetical mechanisms based on the strength of the current evidence.

Growing evidence indicates that gut microbial dysbiosis and microbiota-derived metabolites are associated with and may contribute to the pathogenesis of major neurodegenerative disorders, supporting the concept that microbiota–brain interactions across the lifespan may influence late-life neurological vulnerability. Across Parkinson’s disease (PD), Alzheimer’s disease (AD), and, more preliminarily, amyotrophic lateral sclerosis (ALS), alterations in microbial composition, intestinal barrier integrity, immune signaling, and microbial metabolites converge on shared mechanisms of neuroinflammation, protein aggregation, and neurodegenerative progression along the microbiota–gut–brain axis. These disorders illustrate how microbiota-driven immune, metabolic, and barrier mechanisms may converge on common neurodegenerative pathways (Figure 2). In Parkinson’s disease, human microbiome studies consistently report reduced abundance of Prevotellaceae and enrichment of Enterobacteriaceae, with microbial alterations correlating with disease severity and motor impairment [47]. Reduced Prevotella-mediated mucin metabolism and short-chain fatty acid (SCFA) production may increase intestinal permeability and endotoxin exposure, promoting enteric α-synuclein accumulation and its propagation to the brain via vagal pathways, consistent with the “gut-first” hypothesis of PD pathogenesis [2]. Experimental evidence further supports a causal role of microbiota in PD: α-synuclein-overexpressing mice require the presence of gut microbiota to develop motor deficits and neuroinflammation, while microbial metabolites such as SCFAs can restore pathological phenotypes in germ-free models [48]. These findings suggest that microbiota-dependent immune activation and metabolite signaling may contribute to dopaminergic neurodegeneration. In Alzheimer’s disease, dysbiosis is similarly associated with reduced microbial diversity, depletion of SCFA-producing taxa, and enrichment of pro-inflammatory Gram-negative bacteria, with specific microbial signatures correlating with cerebrospinal fluid amyloid-β and phosphorylated tau biomarkers [49]. Clinical studies integrating microbiome profiling, cytokine expression, and neuroimaging data suggest a microbiota–inflammation–amyloid pathway in which dysbiosis promotes systemic immune activation and neuroinflammatory signaling linked to amyloid deposition [50]. Neuropathological analyses further demonstrate the presence of lipopolysaccharide (LPS) and other bacterial components within AD brain tissue, often colocalizing with amyloid plaques, providing direct evidence that microbial products may contribute to amyloid aggregation and neuroinflammation [51]. Together, these observations support the hypothesis that microbiota-driven immune and metabolic dysregulation may influence key pathological processes underlying Alzheimer’s disease. Compared with PD and AD, evidence for microbiota involvement in ALS remains more limited but increasingly suggestive. Experimental and translational studies indicate that microbial composition and metabolite availability can influence neuroinflammatory responses and disease severity, with taxa such as Akkermansia muciniphila modulating nicotinamide metabolism and motor outcomes in ALS models and patients [52]. Although the mechanistic basis of these associations remains under investigation, these findings raise the possibility that microbiota-derived metabolic pathways may also contribute to motor neuron vulnerability. Collectively, these findings indicate that gut microbial dysbiosis may contribute to neurodegeneration through convergent mechanisms including chronic inflammation, barrier dysfunction, microbial metabolite imbalance, and propagation of pathogenic protein aggregation. Within a lifespan perspective, these mechanisms suggest that microbiota-driven immune and metabolic dysregulation may represent an important biological link between early microbial perturbations and late-life neurodegenerative disease.

## 8. Future Directions and Conclusions

Emerging evidence indicates that the microbiota–gut–brain axis (MGBA) functions as a lifespan regulatory system influencing neurodevelopment, environmental resilience, and susceptibility to late-life neurodegeneration. From this perspective, neurodegenerative disorders may represent the cumulative outcome of microbiota-driven immune and metabolic dysregulation interacting with aging-related vulnerability, rather than isolated late-onset events. A major challenge in the field remains distinguishing causality from association. While experimental models increasingly support mechanistic links between microbial metabolites, immune signaling, and neural dysfunction, much of the human evidence remains correlational. Addressing this gap will require longitudinal cohorts spanning early life to aging, combined with integrative multi-omics approaches, including metagenomics, metabolomics, and host molecular profiling, to identify functional host–microbe pathways and critical windows of vulnerability. From a translational perspective, microbiome-derived features, including taxonomic signatures, inflammatory mediators, and microbial metabolites such as short-chain fatty acids, trimethylamine-N-oxide, and indole derivatives, are being explored as potential biomarkers for disease risk stratification and progression monitoring. Interventions targeting microbiome composition or function, including dietary modulation, probiotics, postbiotics, psychobiotics, and fecal microbiota transplantation, have shown promising but heterogeneous results in experimental studies and early clinical trials. However, inconsistencies across studies, small sample sizes, and variability in study design currently limit the generalizability and clinical translation of these findings. Advancing toward precision microbiome medicine will require accounting for interindividual variability in genetics, immunity, diet, and environmental exposures, alongside the development of systems-biology frameworks capable of integrating host and microbial signals. Although substantial challenges remain, continued integration of mechanistic, clinical, and multi-omics research may ultimately enable microbiome-informed strategies for early prevention and disease modification in neurodegenerative disorders. While summary tables may facilitate comparison across studies, we opted for a narrative synthesis to emphasize mechanistic integration across different levels of evidence.

## Data Availability

No new data were created or analyzed in this study. Data sharing is not applicable to this article.

## Abbreviations

The following abbreviations are used in this manuscript:

MGBA	Microbiota–Gut–Brain Axis
CNS	Central Nervous System
SCFAs	Short-Chain Fatty Acids
HPA	Hypothalamic–Pituitary–Adrenal Axis
LPS	Lipopolysaccharide
BBB	Blood–Brain Barrier
AD	Alzheimer’s Disease
PD	Parkinson’s Disease
ALS	Amyotrophic Lateral Sclerosis
